# Fold Change Detection in Visual Processing

**DOI:** 10.3389/fncir.2021.705161

**Published:** 2021-08-23

**Authors:** Cezar Borba, Matthew J. Kourakis, Shea Schwennicke, Lorena Brasnic, William C. Smith

**Affiliations:** ^1^Department of Molecular, Cell and Developmental Biology, University of California, Santa Barbara, Santa Barbara, CA, United States; ^2^Neuroscience Research Institute, University of California, Santa Barbara, Santa Barbara, CA, United States; ^3^College of Creative Studies, University of California, Santa Barbara, Santa Barbara, CA, United States; ^4^Faculty of Life Sciences and Medicine, King’s College London, London, United Kingdom

**Keywords:** *Ciona*, fold change detection, visuomotor, midbrain, evolution

## Abstract

Visual processing transforms the complexities of the visual world into useful information. *Ciona*, an invertebrate chordate and close relative of the vertebrates, has one of the simplest nervous systems known, yet has a range of visuomotor behaviors. This simplicity has facilitated studies linking behavior and neural circuitry. *Ciona* larvae have two distinct visuomotor behaviors – a looming shadow response and negative phototaxis. These are mediated by separate neural circuits that initiate from different clusters of photoreceptors, with both projecting to a CNS structure called the posterior brain vesicle (pBV). We report here that inputs from both circuits are processed to generate fold change detection (FCD) outputs. In FCD, the behavioral response scales with the relative fold change in input, but is invariant to the overall magnitude of the stimulus. Moreover, the two visuomotor behaviors have fundamentally different stimulus/response relationships – indicative of differing circuit strategies, with the looming shadow response showing a power relationship to fold change, while the navigation behavior responds linearly. Pharmacological modulation of the FCD response points to the FCD circuits lying outside of the visual organ (the ocellus), with the pBV being the most likely location. Consistent with these observations, the connectivity and properties of pBV interneurons conform to known FCD circuit motifs, but with different circuit architectures for the two circuits. The negative phototaxis circuit forms a putative incoherent feedforward loop that involves interconnecting cholinergic and GABAergic interneurons. The looming shadow circuit uses the same cholinergic and GABAergic interneurons, but with different synaptic inputs to create a putative non-linear integral feedback loop. These differing circuit architectures are consistent with the behavioral outputs of the two circuits. Finally, while some reports have highlighted parallels between the pBV and the vertebrate midbrain, suggesting a common origin for the two, others reports have disputed this, suggesting that invertebrate chordates lack a midbrain homolog. The convergence of visual inputs at the pBV, and its putative role in visual processing reported here and in previous publications, lends further support to the proposed common origin of the pBV and the vertebrate midbrain.

## Introduction

The ascidian *Ciona* has served as a valuable model organism both because of its close evolutionary relationship to the vertebrates, and because of its genetic, embryonic, and anatomical simplicity (Satoh, 1994, 2014; Lemaire et al., 2008). Phylogenetically, *Ciona* is a member of the chordate subphylum known as the tunicates. Collectively, the tunicates comprise the closest extant relatives of the vertebrates (Delsuc et al., 2006). The kinship of the tunicates to the vertebrates is evident at all scales – from genomic to anatomical. Particularly striking is the swimming *Ciona* tadpole larva, which highlights both these attributes: vertebrate-like anatomy and simplicity. In common with similarly staged vertebrates, the *Ciona* larva features a notochord running the length of its muscular tail and a dorsal central nervous system (CNS) with a central ventricle. Despite this conserved chordate anatomy, *Ciona* larval organs are composed of very few cells: 40 notochord cells, 36 tail muscle cells, and ∼180 neurons in the CNS (Nicol and Meinertzhagen, 1991; Satoh, 1994). The simplicity of the *Ciona* larval CNS has facilitated the generation of a complete synaptic connectome by serial-section electron microscopy (Ryan et al., 2016).

**FIGURE 1:**
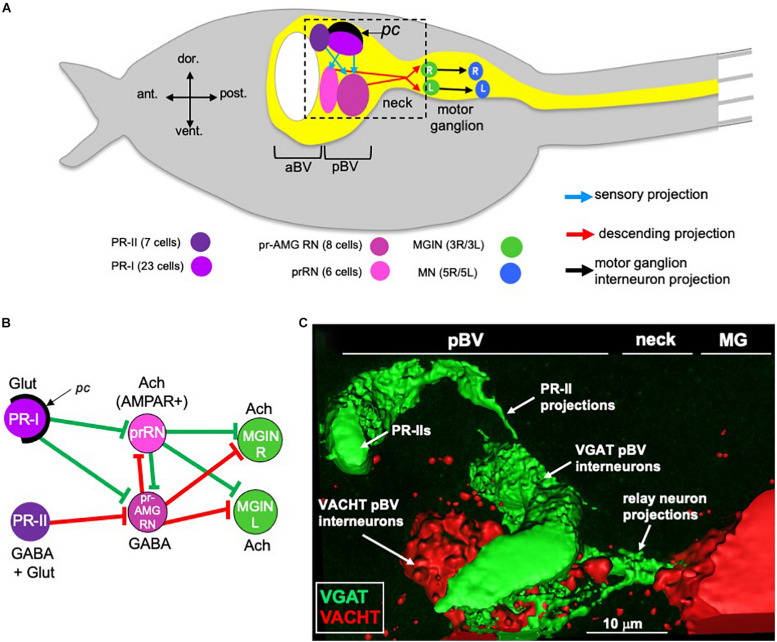
Minimal visual circuitry and anatomy. **(A)** Cartoon of a *Ciona* larva, with emphasis on the anterior (only a small portion of tail is shown at right). Highlighted in the CNS (yellow) are the minimal visuomotor pathways. Cell classes are color coded according to Ryan and Meinertzhagen (2019), and the number of cells in each class are indicated in parentheses. **(B)** Minimal visuomotor circuits. Green lines indicate putative excitatory and red lines putative inhibitory synapses. **(C)** GABAergic PR-II photoreceptors project to GABAergic relay neurons in the pBV. View corresponds approximately to the dashed box in panel **(A)**. pc, pigment cell; aBV, anterior brain vesicle; pBV, posterior brain vesicle; PR, photoreceptor; pr-AMG RN, photoreceptor ascending motor ganglion relay neurons; prRN, photoreceptor relay neurons; MGIN, motor ganglion interneurons; MN, motor neurons; L, left; R, right; Glut, glutamate; Ach, acetylcholine; AMPAR, AMPA receptor; MG, motor ganglion; VGAT, Vesicular GABA transporter; VACHT, Vesicular acetylcholine transporter.

Although small in cell numbers, the *Ciona* larval CNS supports sensory systems that direct a range of complex behaviors. These behaviors include negative gravitaxis, mediated by the otolith organ, mechanosensation, mediated by peripheral touch receptors, and two distinct visuomotor behaviors, mediated by ciliary photoreceptors that cluster into two functional groups in the ocellus organ (Kajiwara and Yoshida, 1985; Svane and Young, 1989; Horie et al., 2008; Ryan et al., 2018; Salas et al., 2018; Bostwick et al., 2020). Figure 1A shows a simplified *Ciona* larva with the visuomotor circuits highlighted. To simplify the diagram, multiple neurons of the same class are grouped together, as are the synaptic connections between them. The first of the two photoreceptor clusters is the PR-I group, which is composed of 23 photoreceptors (Figure 1A). All but two of the PR-I photoreceptors are glutamatergic (Figure 1B). For the other two PR-I photoreceptors, one is GABAergic, and the other is dual glutamatergic/GABAergic (Kourakis et al., 2019). The PR-I photosensory system mediates negative phototaxis with the aid of an associated pigment cell (*pc* in Figure 1) that directionally shades the outer segments of the photoreceptors, allowing larvae to discern the direction of light as they perform casting swims (Salas et al., 2018). The second ocellus photoreceptor cluster, the PR-II group, is composed of seven photoreceptors and is located anterior to the PR-I group (Figure 1A; Horie et al., 2008). The PR-II group does not have an associated pigment cell, and evokes swimming in response to changes in ambient light (dimming), most likely as a looming-shadow escape behavior (Salas et al., 2018). The PR-II group contains a mixture of GABAergic and dual glutamatergic/GABAergic photoreceptors (Figure 1B; Kourakis et al., 2019).

Both the PR-I and PR-II photoreceptors project directly to relay interneurons in the posterior brain vesicle (pBV) (Figure 1A). These relay neurons in turn project primarily to the cholinergic *motor ganglion interneurons* (MGINs) of the motor ganglion (MG) (Figures 1A,B). pBV relay neurons with photoreceptor input fall into two main classes. The six *photoreceptor relay neurons* (prRNs) receive input from the PR-I group (Figures 1A,B). *In situ* hybridization studies indicate that the prRNs are predominantly cholinergic (Kourakis et al., 2019). The other major class of pBV relay neurons with photoreceptor input are the eight *photoreceptor ascending motor ganglion relay neurons* (pr-AMG RNs; Figures 1A,B). The pr-AMG RNs are predominantly GABAergic and receive input from both the PR-I and PR-II groups (Ryan et al., 2016; Kourakis et al., 2019). Thus, the prRNs receive input only from the PR-Is, while the pr-AMG RNs receive input from both photoreceptor groups. Significantly, while both the pr-AMG RNs and the prRNs receive glutamatergic input from the PR-Is, only the cholinergic prRNs express the glutamate AMPA receptor [AMPAR; Figure 1B and (Kourakis et al., 2019)]. Moreover, treatment with the AMPAR antagonist perampanel blocks negative phototaxis while not disrupting the light-dimming response (Kourakis et al., 2019). Thus the minimal circuit for negative phototaxis appears to involve the glutamatergic photoreceptors stimulating the cholinergic prRNs, which then project to the MG to stimulate the cholinergic MGINs. The MGINs then activate the cholinergic motor neurons to evoke swimming (Figures 1A,B). The significance of the non-AMPAR glutamatergic input to the pr-AMG RNs is explored in the present study.

The circuit logic for the PR-II mediated dimming response is more complex. GABAergic projections from the PR-IIs are targeted exclusively to the predominantly GABAergic pr-AMG RNs (Figures 1B,C). This arrangement led to a disinhibitory model for the light-dimming response (Kourakis et al., 2019). In this model, swimming is actively inhibited by pr-AMG RN input to the MGINs, unless they are themselves inhibited by the GABAergic photoreceptors (Figure 1B). Tunicate photoreceptors, like their vertebrate counterparts, are hyperpolarizing (Gorman et al., 1971), and thus dimming is expected to increase their GABA release. Moreover, behavioral analyses with the GABA antagonist picrotoxin, as well as in the mutant *frimousse*, in which the photoreceptors are absent due to a transfating of the anterior brain vesicle (aBV) to epidermis (Hackley et al., 2013), indicate that swimming behavior in the untreated, wild-type larva is constitutively inhibited, consistent with the disinhibition model (Kourakis et al., 2019).

Both *Ciona* visuomotor behaviors are responses to changing illuminations, whether it be decreased ambient illumination for the PR-II circuit, or directional photoreceptor shading in the PR-I circuit. For both visuomotor circuits to function in changing illumination conditions, dynamic visual processing is required. We report here that the *Ciona* larval CNS processes visual inputs to detect fold change (FC) differences. In fold change detection (FCD), the response depends only on the relative change in input, and not on the absolute change (Adler and Alon, 2018). FCD allows a sensory system to give a consistent behavioral response to the same relative change, independent of the ambient conditions, while suppressing noise. Moreover, we present evidence that the circuits for FCD are distinct from the adaptive mechanisms of the photoreceptors, and instead appear to be present in the complex of synaptic connectivity between relay neurons in the pBV. Finally, we note that the convergence of anatomical, molecular, connectomic and behavioral data point to the *Ciona* pBV as sharing homology to the vertebrate midbrain, suggesting a common origin of visual processing centers, such as the vertebrate optic tectum (OT) (Knudsen, 2020), and thus this function may predate the split of the tunicates and the vertebrates.

## Results

### Larval Visuomotor Behaviors Display Fold Change Detection

**FIGURE 2:**
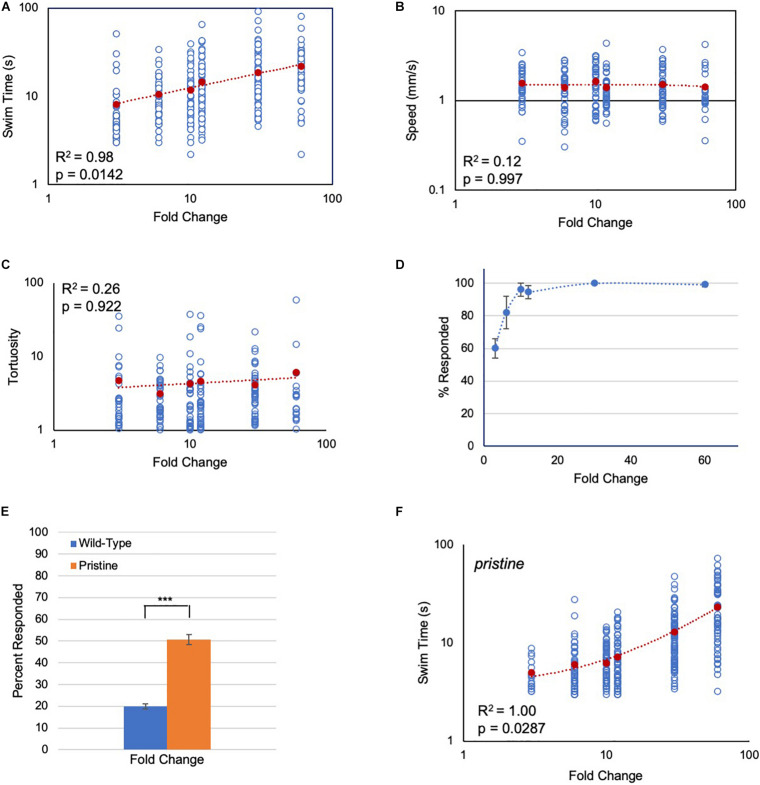
Response of *Ciona* larvae to fold change light dims. **(A)** Larval swim times increase as a power function in response to increased fold change light dimming (3-fold to 60-fold; log/log plot shown). All data points (blue circles) and averages (red circles) are shown [same for panels **(**B**,**C**,**F**)**]. See Movie 1 for representative results. **(B)** Swim speed is constant across fold change dimming series (log/log plot shown). **(C)** Swim tortuosity is constant across fold change dimming series (log/log plot shown). **(D)** The percent of larvae responding as a function of fold change dimming. Shown in graph are the averages from three recordings (± S.D.). **(E)** Percent of larvae responding to a six-fold dim at low illumination conditions (30 lux to 5 lux). *n* = 76 and 131, for wild-type and *pristine*, respectively (*** = *p* < 0.001; Wilcoxon Rank-Sum test). **(F)** Larval swim times of homozygous *pristine* mutants increase as a linear function in response to increased fold change light dimming (3-fold to 60-fold; log/log plot shown). See Supplementary Figure 1 for sample sizes, average values, and statistical analyses. All larvae were observed for 2 min after dimming. The *p*-values shown were determined from a Kruskal-Wallis test.

In the first set of experiments, the response of *Ciona* larvae to a light-dimming series from 3-fold (600 lux to 200 lux) to 60-fold (600 lux to 10 lux) was assessed. Several parameters of the dimming-induced swims were measured: the percent of larvae responding to dimming and their reaction time, as well as the duration, speed, and tortuosity of swims. Tortuosity measures the deviation from straight-line swim trajectories (Salas et al., 2018). Movie 1 shows representative responses to 3-, 10-, and 60-fold dims. Of these parameters, induced swim duration showed a positive relation to increased FC (Figure 2A), while speed and tortuosity were constant across the series (Figures 2B,C; Supplementary Figure 1). The percent of larvae responding to dimming also did not track with the FC series. The percent responding increased initially at the lowest FCs, but plateaued at around 10-fold with ∼100% of larvae responding (Figure 2D).

While the dimming response is an output of the PR-II circuit, the PR-I negative phototaxis circuit depends on larvae detecting changing illumination as they perform casting swims. Although we have reported that *Ciona* larvae are able to successfully navigate in a wide range of ambient lighting conditions (Salas et al., 2018), the phototaxis assay would not permit precise control of the amount of light the PR-I photoreceptors were receiving, making it difficult to assess their responses to FC stimuli. To circumvent this problem, we used the loss-of-pigmentation mutant *pristine* (*prs*) to assess the PR-I photoreceptors, as we have done previously (Salas et al., 2018; Kourakis et al., 2019). In larvae homozygous for *prs*, the PR-I photoreceptors respond to ambient light changes because they are no longer shielded by the pigment cell; in other words, changes in ambient light mimic casting swims (Salas et al., 2018). While both the PR-I and PR-II photoreceptors would be stimulated by dimming in *prs* mutants, there are more PR-I photoreceptors than PR-II (23 versus 7), and the PR-I output appears to predominate (Salas et al., 2018). One way this is evident is that the dimming-evoked swims of *prs* mutants are straight, as are phototaxis swims, rather than highly tortuous, as are dimming-induced swims (Salas et al., 2018). To validate this further, we find that *prs* larvae are more sensitive to dimming at low-light conditions than wild-type larvae (Figure 2E), consistent with the behavioral output from *prs* mutants primarily reflecting the output from the PR-I circuit.

When the FC dimming series was performed on *prs* larvae, we again observed a positive relationship of swim time to FC, but with a significantly different shape to the response curve (Figure 2F and Supplementary Figure 1C; *p* = 0.0007, Friedman’s test). Modeling indicates that a number of different circuit motifs, including the incoherent type-1 feedforward loop (I1FFL) and the non-linear integral feedback loop (NLIFL), can generate FCD outputs (Adler et al., 2017). Moreover, different FCD circuit motifs can generate different response curves (e.g., linear or power), meaning that the response curves can be diagnostic of the underlying circuit architecture (Adler and Alon, 2018). For wild-type *Ciona* larvae, the curve of swim time versus FC was found to best fit a power function, with *R*^2^ = 0.98 (Figure 2A). While a log function also fit this curve with *R*^2^ = 0.98, the Bayesian information criterion (BIC, see section “Materials and Methods”) for a power relationship had the lower score, indicating a better fit (−13 and 17 for power and log, respectively; *R*^2^ = 0.87 for linear; Supplementary Figure 1A). The best fitting model for the *prs* swim time responses was a linear curve having an *R*^2^ value equal to 1.00 (Figure 2F). When the *prs* data were fitted to power and log functions, the *R*^2^ values were 0.92 and 0.81, respectively (Supplementary Figure 1B). In summary, wild-type and *prs* larvae both show a positive relationship between FC dimming and swim time, although with different response curves, suggesting that different FCD circuits may be responsible.

### Validation of FCD Behavior

**FIGURE 3:**
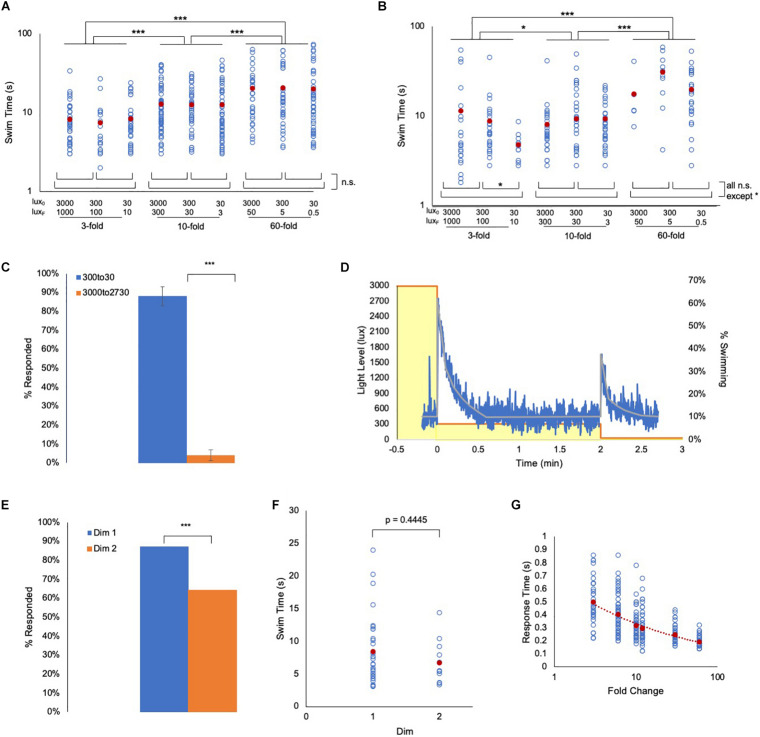
*Ciona* fold change detection response. **(A)** *Ciona* larvae swim times show scale-invariance to light dimming across three orders of magnitude. Lux_0_: initial illumination level in lux; Lux_*F*_: illumination after dim in lux (log/log plot shown). **(B)** Same light-dimming series as in panel A, but with *pristine* mutants (log/log plot shown). **(C)** The *Ciona* light-dimming response follows Weber’s law. Shown are the percent of larvae responding to 270 lux dimming from the initial conditions of 300 lux or 3000 lux (*n* = 776 and 443; respectively). **(D)** *Ciona* larvae show absolute adaptation to light-dimming. Larvae were exposed to two light dimmings separated by 2 min (3000 lux to 300 lux, and 300 lux to 30 lux; yellow boxes). The blue line shows the percent of larvae swimming at 1 s time intervals (*n* = 91–320). The gray line is the average at each time point. See also Movie 2. **(E)** For data shown in panel **(D)**, a higher percentage of larvae responded to the 3000 to 300 lux dim (Dim 1) than to the 300 to 30 lux dim (Dim 2). **(F)** For the experiment shown in panel **(D)**, the swim times induced by the 3000 to 300 lux dim (Dim 1) were not different from the 300 to 30 lux dim (Dim 2). **(G)** Plot of the reaction time versus fold change. This is defined as the time point at which swimming was first detected following dimming. For panels **(A,B,F,G)**, all data points (blue circles) and averages (red circles) are shown. See Supplementary Figure 2 for full data and statistical analyses. (*** = *p* < 0.001; * = *p* < 0.05; n.s. = not significant).

Fold change detection mechanisms, while incorporating widely observed phenomena such as adaptation and log transformation, have distinct attributes – the most important being scale-invariance (Goentoro et al., 2009; Shoval et al., 2010; Kamino et al., 2017). With scale-invariance the output depends only on the FC, not on the absolute magnitude of the stimulus. We find scale-invariance holds true for the *Ciona* visuomotor response across at least three orders of magnitude. To assess scale-invariance, wild-type and *prs* larvae were exposed to series of 3-, 10-, and 60-fold dims, but from starting intensities of 3000, 300, and 30 lux (e.g., the 10-fold dims were 3000 to 300 lux, 300 to 30 lux, and 30 to 3 lux). We observed that the swim time responses of both wild-type and *prs* larvae were not significantly different within a FC, irrespective of the magnitude of illumination, but were significantly increased as FC increased (Figures 3A,B and Supplementary Figures 2A,B).

Integral to FCD behavior is Weber’s law (i.e., the change in stimulus needed to elicit a response is proportional to the absolute value of the original stimulus) (Adler and Alon, 2018). To demonstrate this directly in *Ciona* visuomotor behavior, larvae that were adapted to either 3000 lux or 300 lux were then dimmed by 270 lux (i.e. 2730 or 30 lux, respectively). In those larvae adapted to 3000 lux, we observed no response to the dimming, while in the larvae adapted to 300 lux, we observed vigorous swimming in nearly all larvae (Figure 3C). Another property of FCD systems is exact adaptation [i.e., the system returns to the baseline state even when the modulated stimulus persists at the new state (Shoval et al., 2010)]. To investigate this, larvae were initially adapted to 3000 lux, which was then dimmed to 300 lux and held at this level for 2 min. The illumination was then dimmed a second time, to 30 lux (Movie 2). Figure 3D shows a plot of swimming activity of the larvae as illumination levels change. We observed that the larvae respond robustly to the first 10-fold dim (3000 to 300 lux, yellow boxes in Figure 3D), but stop swimming after approximately 30 s. The majority of the larvae were then stationary until the second 10-fold dimming (300 to 30 lux). While a lower percent of larvae responded to the second dim than to the first (87% vs. 64%, Figure 3E), no difference in the average swim times of responding larvae was observed (Figure 3F). Finally, another predicted property of FCD systems is that the reaction time should be inversely proportional to the FC (Adler and Alon, 2018), which we observed as a power-slope increase in reaction time as the FC decreased (Figure 3G and Supplementary Figures 2C,D).

### Pharmacological Modulation of FCD Circuits

In order to investigate the FCD circuits in the *Ciona* visuomotor system we used pharmacological agents to modulate the response. For the PR-II dimming-response pathway (Figure 1B), we had previously shown that the AMPAR antagonist perampanel does not disrupt the dimming response, but does block PR-I mediated phototaxis (Kourakis et al., 2019). While these results, and the circuit logic of the connectome, indicate that dimming-induced swimming is mediated by GABAergic inhibition of the pr-AMG RNs, there are also extensive synaptic connections between the pr-AMG RNs and the AMPAR-expressing prRNs (Figure 1B and Ryan et al., 2016). Synaptic activity between these two classes of interneurons, one primarily excitatory and the other primarily inhibitory, may play a role in FCD. To assess this we used the AMPAR agonist AMPA, reasoning that while perampanel would simply block glutamatergic input from the PR-Is to the prRNs, and thus should not directly impact the interactions of GABAergic and cholinergic pr-AMG RNs and prRNs (see Figure 1B), AMPA, as an agonist, should directly alter the state of the prRNs and could reveal a role in FCD.

**FIGURE 4:**
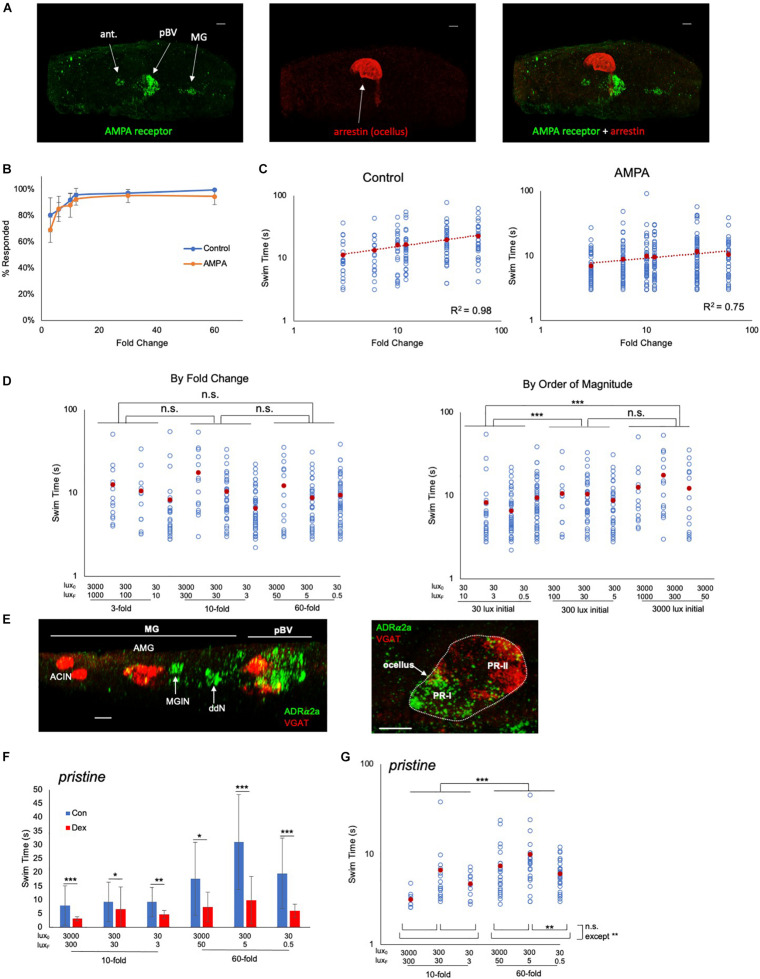
Pharmacological modulation of behavioral responses. **(A)** AMPA receptor expression detected by *in situ* hybridization (green; left panel). Photoreceptors were detected by immunostaining for Arrestin detected by immunostaining (red; middle panel). Dorsal view, anterior to left. Right panel shows a merged view. Scale bars are 10 μm.**(B)** Percentage of control and AMPA-treated larvae responding to the indicated fold change light dimmings series. The averages from three recordings are shown (± S.D.). **(C)** Swim times of control and AMPA-treated larvae in the indicated fold change dimming series. The *R*^2^ valves for the curves are indicated. **(D)** AMPA-treated larvae do not show scale-invariance to fold-change dims of different magnitudes (left panel; compare to controls in Figure 3A). Right panel shows the same data sorted by magnitude of initial illumination (lux_0_). **(E)** *In situ* hybridization for ADRα2a (green) and VGAT (red). Scale bars are 10 μm. **(F)** Swim times for control (“con”; blue bars) and dexmedetomidine-treated (“dex”; red bars; 20 μM) *pristine* larvae. Shown are the responses to 10- and 60-fold dimming over three illumination ranges. Error bars represent standard deviations. **(G)** Dexmedetomidine-treated *pristine* larvae respond to increased fold change dimming with increased swim times. For panels **(C,D,F,G)** all data points (blue circles) and averages (red circles) are shown and are plotted log/log. (* = *p* < 0.05; ** = *p* < 0.01; *** = *p* < 0.001; n.s. = not significant; Wilcoxon Rank-Sum test). For full data and statistical analyses see Supplementary Figures 3–5. ant., antenna cells; pBV, posterior brain vesicle; MG, motor ganglion; PR, photoreceptor; MGIN, motor ganglion interneurons; VGAT, Vesicular GABA transporter; ddN, descending decussating neuron; AMG, ascending motor ganglion interneuron; ACIN, ascending contralateral inhibitory neurons; ADRα2a, α2 adrenoreceptor.

We observed that AMPAR is expressed, in addition to the pBV, in the antenna cells and the MG (Figure 4A). Significantly, we did not observe expression in the photoreceptors, showing that any observed modulation of FCD behavior was taking place outside of the photoreceptors. Moreover, neither the antenna or MG cells are likely contributors to the FCD processing. Firstly, our previous observations showed that blocking AMPARs with perampanel did not disrupt the antenna cell-mediated gravitaxis at 25 hpf (Bostwick et al., 2020), and secondly, the MG expression of AMPAR is restricted to the left set of the bilaterally paired MGINs (Kourakis et al., 2021). Thus, while we might expect the AMPA to induce or potentiate swimming (possibly via the MGINs or antenna-cells), we observed no difference in the percentage of control and AMPA-treated (500 μm) larvae responding to a fold-dimming series (Figure 4B; Supplementary Figure 3). However, a plot of swim time versus FC (Figure 4C) shows the slopes of the two curves were significantly different (R^2^ of 0.98 for control, and 0.75 for AMPA-treated; *p* = 0.0002, Friedman’s test), with AMPA-treated larvae showing much less increase in swim time as the FC series increased. In a second set of experiments in which control and AMPA-treated larvae were assessed against a series of identical FCs but of different magnitudes, the disruption to the FCD mechanism was evident (Figure 4D). When the data were grouped according to FC, no significant differences in swim times were found between FCs (Figure 4D, left panel), unlike in untreated larvae (Figure 3A). However, when the data were grouped by magnitude of the starting illumination, larvae appeared to respond according to the magnitude of illumination, rather than FC (Figure 4D, right panel). For example, larvae assessed from a starting illumination of 30 lux had shorter swims than larvae assessed from a starting illumination of 300 lux, independent of the FC. This was true for comparisons across all starting illuminations, with the exception of 300 lux versus 3000 lux.

We assessed a second pharmacological agent, the adrenergic agonist dexmedetomidine, which has previously been shown to modulate the dimming response of *Ciona* (Razy-Krajka et al., 2012), to determine whether it also disrupted FCD. The target of dexmedetomidine, the α2 adrenoreceptor ADRα2a, was shown with use of a *cis*-regulatory element reporter construct to be expressed in VACHT-positive neurons of the pBV. By *in situ* hybridization analysis we confirmed expression in the pBV (Figure 4E, left panel), as well as two groups of the neurons in the MG that we have tentatively identified as ddNs and MGINs based on their locations to each other and the AMG cells. We also observed expression in the photoreceptors of the ocellus (Figure 4E, right panel). The strongest ADRα2a expression was in the posterior, non-VGAT expressing, photoreceptors, which corresponds to the PR-I group (Kourakis et al., 2019). Consistent with the previous report (Razy-Krajka et al., 2012), we observed that dexmedetomidine treatment decreased swim times in response to dimming for both wild-type (Supplementary Figure 4) and *prs* larvae (Figure 4F; and Supplementary Figure 5). The dimming response in wild-type larvae was greatly reduced by dexmedetomidine, and a response to different FC dimmings was most evident at the highest magnitude illumination level (3000 lux; Supplementary Figures 4B,C). By contrast, dexmedetomidine-treated *prs* larvae, despite being significantly inhibited by dexmedetomidine, showed more robust responses at all illumination levels for 10- and 60-fold dimming (Figure 4G). At three-fold dimming too few *prs* larvae responded for the analysis (Supplementary Figure 5).

In summary, results with dexmedetomidine-treated larvae contrast with those from AMPA-treated larvae. AMPA treatment disrupts FCD, but not the ability of the larvae to respond to dimming. Moreover, AMPA-treated larvae appear to respond to the magnitude of the illumination, not the FC. By contrast dexmedetomidine treatment results in an overall decrease in responsivity to dimming, but we still see evidence of FCD. These results indicate that the FCD element of the *Ciona* visual response circuits can be separated from the detection of illumination. In addition, the fact that the FCD element can be disrupted by AMPA indicates that the synaptic activity of the circuit is essential for FCD, which contrasts with other characterized FCD mechanisms, in which signal transduction pathways appear sufficient to account for FCD (Adler and Alon, 2018).

### Is the pBV a Homolog of the Vertebrate Midbrain?

The pBV is the primary recipient of projections from the ocellus, otolith, and coronet cells, and a subset of peripheral neurons (Ryan et al., 2016). Relay neurons within the pBV then project posteriorly through the neck to the MG. No other region of the *Ciona* CNS has this convergence of sensory inputs and descending interneuron projections. Results presented here, as well as published studies (Bostwick et al., 2020), point to the pBV as a sensory processing and integrating center. Thus in many ways the function of the pBV resembles that of the vertebrate midbrain visual processing centers, including the optic tectum (Knudsen, 2020). The resemblance of the pBV to the vertebrate midbrain extends to the *Ciona* CNS anatomy as well. In particular, the pBV is located immediately anterior to the neck region which, based on gene expression and the fact that it forms a constriction in the CNS, is thought to have homology to the vertebrate midbrain-hindbrain junction (Ikuta and Saiga, 2007). Despite these anatomical similarities, it has been widely speculated that tunicates either do not have, or have lost a midbrain homolog. These reports are based on the expression patterns of several genes that do not match the expression of their vertebrate orthologs. For example, the gene DMBX, which plays an essential role in vertebrate midbrain development, is not expressed anterior to the MG in *Ciona* (Takahashi and Holland, 2004; Ikuta and Saiga, 2007). In addition, the tunicate *Oikopleura dioica* (Class Larvacea) does not express the genes *engrailed* or *pax2/5/8* anterior to its hindbrain, suggesting that larvaceans lack a midbrain (Cañestro et al., 2005). However, these studies were limited to a few genes, and were performed before the connectivity of the pBV was made apparent by the publication of the connectome. Moreover, as presented below, a wider view of neural genes shows extensive gene expression conservation between the pBV and the vertebrate midbrain.

**FIGURE 5:**
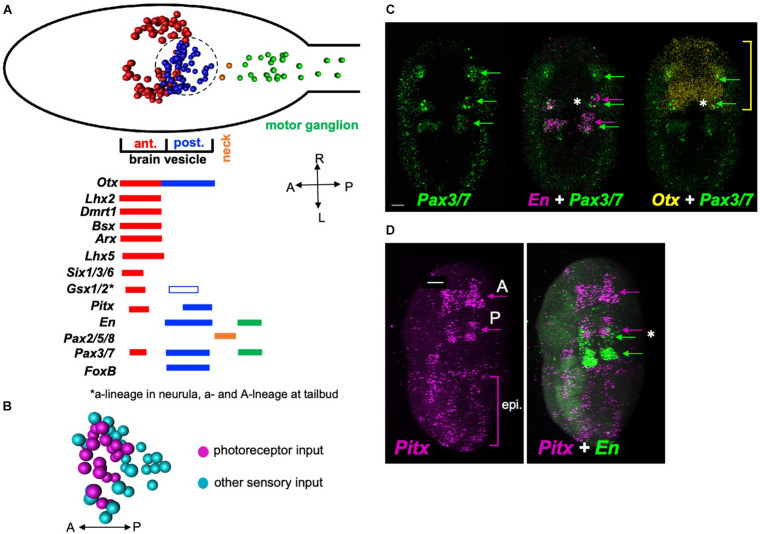
Gene expression in the *Ciona* posterior brain vesicle suggests homology with the vertebrate midbrain. **(A)** Top: Diagram of the *Ciona* larval CNS with the major brain regions indicated in color. The centroids of neurons are shown (from Ryan et al., 2016). Bottom: summary of embryonic gene expression patterns marked by corresponding larval CNS domains. Domains in red show expression for orthologs to vertebrate forebrain markers, blue to midbrain, orange to MHB, and green to spinal cord. **(B)** Spatial distribution of relay neuron types in the pBV. Region shown corresponds to the circled area in panel **(A)** (centroids shown). **(C)** *In situ* hybridization for *Pax3/7*, *En*, and *Otx* in early tailbud *Ciona* embryos. The anterior domain of *En*, which marks the presumptive pBV, overlaps with *Pax3/7* (asterisk middle panel), and *En* (asterisk right panel). Yellow bracket shows anterior-posterior extent of *Otx* expression. **(D)** *In situ* hybridization for *Pitx* and *En*. *Pitx* is expressed in anterior and posterior domains in early tailbud embryos. The posterior domain of *Pitx* overlaps with the anterior *En* domain in the pBV (asterisk right panel). Consistent with previous reports, labeling was also observed in the epidermis. Green arrows in panels **(C)** are indicating domains of *Pax3/7* expression in the developing central nervous system. In panel **(D)**, green arrows indicate domains of *engrailed* expression, and magenta arrows indicate domains of *Pitx* expression the central nervous system. Asterisk indicates overlapping expression of *Pitx* and *engrailed*. A, anterior; P, posterior; epi, epidermis. Anterior is to the left for panel A and D, and to the top for B and C. Scale bars are 10 μm.

The *Ciona* BV is divided into distinct anterior and posterior domains that derive from invariant cell lineages arising at the 8-cell stage, with the anterior BV (aBV) descending from the a-lineage, and the pBV from the A-lineage (Figure 5A, red and blue cell centroids, respectively) (Nishida, 1987; Nicol and Meinertzhagen, 1988). Moreover, the distribution of neuron types is sharply demarcated by this boundary, with the relay neurons, which uniquely project from the BV to the MG, being found only in the pBV. The relay neurons are themselves segregated within the pBV, with those receiving photoreceptor input clustered anteriorly (Figure 5B). The gene *Otx*, which is expressed in the forebrain and midbrain of vertebrates (Boyl et al., 2001), is expressed in *Ciona* in both the aBV and pBV (Imai et al., 2002; Hudson et al., 2003; Ikuta and Saiga, 2007), while a number of the vertebrate forebrain markers are expressed only in the aBV lineage. This includes the genes *Dmrt1* (Tresser et al., 2010; Wagner and Levine, 2012; Kikkawa et al., 2013), as well as *Lhx5*, *Six3*, and *Gsx2* (Mazet et al., 2005; Moret et al., 2005; Esposito et al., 2017; Reeves et al., 2017), all of which play essential roles in vertebrate forebrain development (Toresson et al., 2000; Lagutin, 2003; Peng, 2006; Kirkeby et al., 2012). While *Gsx2* is also expressed in the pBV, this is only in later stages of development (tailbud stages). Additional vertebrate forebrain markers expressed exclusively in the aBV lineage include *Lhx2/9*, *Bsx* (or *Bsh*) and *Arx* (or *Aristaless*) (Cao et al., 2019). In vertebrates these genes are reported to play essential roles in cortex development (Shetty et al., 2013; Roy et al., 2014; Marsh et al., 2016; Schredelseker et al., 2020).

By contrast, the pBV expresses a number of genes associated with the vertebrate midbrain. This includes the A-lineage specific marker *FoxB1* (or *Ci-FoxB*) (Moret et al., 2005; Oonuma et al., 2016), which in vertebrates plays a role in midbrain development (Wehr et al., 1997). The development of the vertebrate optic tectum, a midbrain structure, requires the co-expression of *Pax3*, *Pax7*, *Otx2*, and *En* (Matsunaga et al., 2001; Thompson et al., 2008). The *Ciona engrailed* ortholog is expressed in two domains embryologically: posteriorly in the MG and anteriorly in the pBV (Imai et al., 2002; Ikuta and Saiga, 2007). We observed overlapping expression of *Pax3/7*, *En*, and *Otx2* in the developing pBV of early tailbud embryos (Figure 5C, asterisk). Finally, *Pitx* has a well-defined role in vertebrate midbrain development (Luk et al., 2013). We observed *Ciona Pitx* expression at early tailbud stage in a posterior domain that overlaps with pBV *engrailed* expression (Figure 5D, asterisk), as well as in an anterior domain that appears to correspond to the aBV expression reported in older embryos and larvae (Christiaen et al., 2002). In addition, diffuse epidermal labeling was observed, as reported previously (Boorman and Shimeld, 2002). The pBV is bounded posteriorly by expression of *Pax2/5/8* in the neck cells, indicating shared homology with the vertebrate MHB (Ikuta and Saiga, 2007; Figure 5A).

These expression pattern results show that the BV has distinct anterior and posterior expression domains, with the anterior domain expressing genes known to be expressed in the developing vertebrate forebrain, and the posterior domain expressing genes associated with the developing vertebrate midbrain. These observations do not agree with previous reports that suggest the entire BV is homologous to the vertebrate forebrain (and that a midbrain homolog is absent). Thus the convergence of gene expression, anatomical, connectivity and functional data all point to the pBV as sharing a common origin with the vertebrate midbrain.

## Discussion

The behavioral studies presented here demonstrate that *Ciona* larvae transform visual input to detect FCs. The utility of this behavior is clear: in negative phototaxis, larvae discern the direction of light via casting swims, and it is the change in illumination falling on the PR-Is as larvae turn away from the light that is the cue to swim. FCD ensures that the casting mechanism functions in the wide range of ambient light conditions that larvae are likely to encounter, and that the response is invariant to the scale of the input. The function of FCD to the dimming response is similar. In the absence of FCD, the change in illumination caused by the same looming object that appeared to be a threat in one ambient light condition, might not be in another. FCD ensures that the response varies as a function of the relative shading caused by the looming object. Comparison of the response curves of wild-type and *prs* larvae to a FC series indicates that different mechanisms are operable in the two pathways, although it is not known if these stimulus-response relationships are each better suited for the type of behavior being mediated.

A number of cellular signaling systems have been shown to give FCD responses to extracellular cues, including those in bacterial chemotaxis and growth factor signaling in mammalian cells and embryos (Goentoro et al., 2009; Shoval et al., 2010; Adler and Alon, 2018; Lyashenko et al., 2020). Modeling has identified several classes of biological circuits that can generate FCD responses (Goentoro et al., 2009; Hironaka and Morishita, 2014; Adler et al., 2017), although in many examples of FCD the biological circuits remain to be determined. For the FCD responses described here, the combination of the connectomic, behavioral and pharmacological data point to candidate FCD circuits. In particular, the results with the drug AMPA, in which the larvae remain responsive but no longer show FCD, indicate that neural circuits, and not only intracellular signal transduction pathways can generate FCD responses. Nevertheless, the *Ciona* photoreceptors themselves, because of their presumed adaptive properties, almost certainly play a role in processing the visual inputs by extending the dynamic range. Vertebrate photoreceptors, in particular cones, have properties that might by themselves generate a FCD output, including adaptation and adherence to Weber’s Law (Burkhardt, 1994). However, to our knowledge, FCD by vertebrate photoreceptor phototransduction machinery has not been directly assessed, and modeling suggests that adaptation and adherence to Weber’s law alone are not sufficient to give FCD (Shoval et al., 2010). Moreover, much of the adaptive properties of the vertebrate visual system arise not only from the transduction mechanism inherent to the photoreceptors, but also from the neural circuitry in the vertebrate retina (Dunn et al., 2007).

While the presence of AMPA receptors in both the pBV and the MG complicates the identification of candidate FCD circuits, the properties of the pBV make it a more likely candidate for containing the FCD circuits. The *Ciona* connectome shows that the pBV is unique among the brain regions in receiving direct input from several sensory systems including the photoresponsive ocellus, the gravity sensitive otolith, the dopaminergic coronet cells, and a subset of the peripheral sensory neurons (Ryan et al., 2016, 2018). Relay neurons from the pBV then project to a common set of six secondary cholinergic interneurons (MGINs) and ten motor neurons in the MG (Figure 1A). This circuit architecture of converging sensory inputs implicates the pBV as a site of sensory integration and processing. For example, *Ciona* larvae integrate visual and gravitactic inputs into a single behavior consisting of upward swimming in response to light dimming (Bostwick et al., 2020). The projections from these two sensory systems converge and are interconnected at the pBV. While the two photoreceptor pathways in *Ciona* appear to operate in parallel, they too converge at the pBV, but with different circuit architectures (Figure 1) and logic (Kourakis et al., 2019). Significantly, a closer examination of the interneurons in the pBV which receive input from the two photoreceptor systems suggests circuits that can account for the different stimulus-response curves (Figure 2).

**FIGURE 6:**
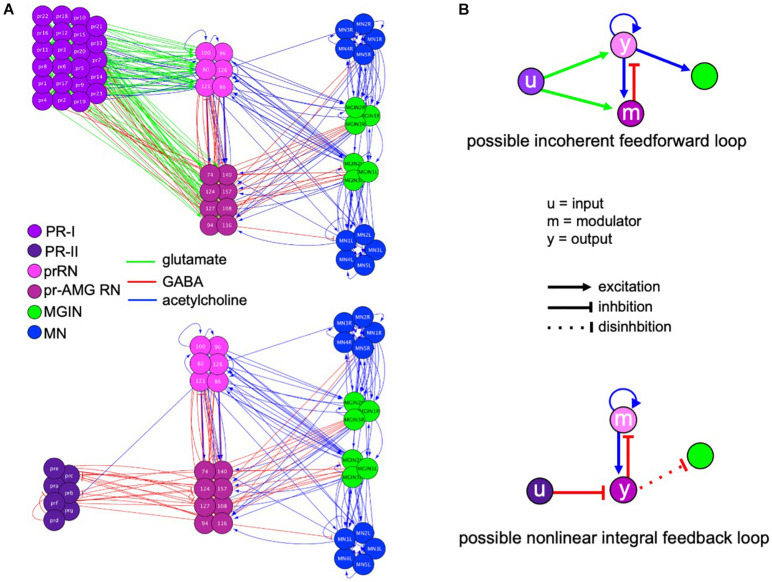
Visuomotor circuits and putative fold change detection circuits. **(A)** Full circuits for the PR-I (top) and PR-II (bottom) pathways from the *Ciona* connectome (Ryan et al., 2016). Neurotransmitter use for synaptic connections (lines) is based on Kourakis et al. (2019). PR, photoreceptor; prRN, photoreceptor relay neuron; pr-AMG RN, photoreceptor ascending motor ganglion relay neurons; MGIN, motor ganglion interneurons; MN, motor neurons. **(B)** Simplified circuits for the PR-I (top) and PR-II (bottom) pathways derived by combining like cells and assigning valence of synapses (excitatory or inhibitory) based on consensus for that cell type. Nodes are labeled according to the proposed function (i.e., input modulator, and output). Colors of neuron classes are according to Ryan and Meinertzhagen (2019).

Figure 6A shows the full PR-I and PR-II visuomotor circuits, as given by the *Ciona* connectome (Ryan et al., 2016) with superimposed putative neurotransmitter types, as deduced by *in situ* hybridization (Kourakis et al., 2019). When the PR-I and PR-II circuits are simplified by combining cell types and synaptic connections, two plausible FCD circuits are evident, with prominent roles played by the pBV relay neurons (Figure 6B). The PR-I circuit contains a putative incoherent feedforward loop (IFFL), while the PR-II circuit contains a putative non-linear integral feedback loop (NLIFL). Experimental and modeling studies show that these two circuits will give very different response curves to a FC series (Adler and Alon, 2018). The NLIFL gives a power relationship, as we observed for wild-type larvae (Figure 2A). The proposed PR-I IFFL circuit differs from the widely studied type-1 IFFL in having an additional excitatory interaction from the output (y) to the modulator (m) (top, Figure 6B). While computer modeling of circuit motifs indicates that this motif should give FCD (Adler et al., 2017), the relationship between FC and output has not been modeled. Nevertheless, the presence of two different FCD motifs in the PR-I and PR-II circuits is consistent with the observed differences in behavioral responses to the FC series. We note that in the two proposed FCD circuits in Figure 6B, the RN types exchange roles as output (y) and modulator, suggesting an economy of neuron use.

The proposed NLIFL motif in the PR-II circuit, being disinhibitory, has circuit components that are switched compared to conventional NLIFL motif (bottom, Figure 6B). We hypothesize that this circuit would have the same sensory processing function because the key elements are all present, albeit with opposite polarity. In other words, in disinhibitory circuits the inhibition of the output evokes the response (i.e., swimming). In a conventional circuit the modulator inhibits the output. However, in the disinhibitory circuit the modulator activates the output. The result of both is to decrease the response, and the decrease is proportional to the input, which is a key feature of FCD circuits. Consistent with the AMPAR-expressing cholinergic prRNs acting as the modulator in the disinhibitory circuit, addition of AMPA disrupts FCD (Figure 4).

Unlike in the pBV, analysis of the MG circuitry did not reveal plausible FCD circuits. The MG is dominated by excitatory cholinergic interneurons and motor neurons, while inhibitory neurons, which would be an essential modulatory element of any likely FCD circuit, are limited to the GABAergic AMG neurons, which receive no descending input, directly or indirectly, from the photoreceptors or the BV, and the glycinergic decussating ACINs, which likely play a role in the central pattern generator, not visual processing (Nishino et al., 2010; Ryan et al., 2016; Kourakis et al., 2019). In addition, the PR-I and PR-II circuits project from the pBV to a common set of MG interneurons, making it unlikely that this brain region would be responsible for the different FC response curves for the PR-I and PR-II circuits.

The homology of the various anterior-to-posterior domains of the tunicate larval CNS to those of their vertebrate counterparts is still a matter of discussion [reviewed in Hudson (2016)]. Of particular controversy is the presence of a midbrain homolog, with gene-expression data used both to argue for Wada et al. (1998); Imai et al. (2002), and against (Cañestro et al., 2005; Ikuta and Saiga, 2007) homology. It has been proposed that the midbrain is a vertebrate invention (Takahashi and Holland, 2004), although evidence for a midbrain in amphioxus argues against this (Holland, 2015; Lacalli, 2018). Nonetheless, the “neck” region of the *Ciona* CNS has apparent conserved gene expression, and thus presumed homology, to the vertebrate midbrain/hindbrain junction (Imai et al., 2009). However, the Otx-expressing CNS region anterior to the neck (called variously the *sensory vesicle* or the *brain vesicle*), has been equated with either the vertebrate forebrain in its entirely, or with separate forebrain and midbrain domains (Wada et al., 1998; Imai et al., 2002, 2009). Figure 5 shows nine orthologs of vertebrate forebrain markers that are expressed in the aBV, but not in the pBV. This is compelling evidence that the entire brain vesicle does not have homology to the forebrain. Thus the pBV is sandwiched between a region anterior to it that expresses forebrain markers, and a region posterior to it that expresses midbrain/hindbrain junction markers. Figure 5 also presents five genes that are expressed in both the vertebrate midbrain and in the posterior brain vesicle of *Ciona*. The *Ciona* connectome provides added perspective to this issue: the pBV, which is distinct from the aBV in a number of ways, including its cell lineage (Cole and Meinertzhagen, 2004), is the principal target of input from the visual system, as is the midbrain, particularly in lower vertebrates (Knudsen, 2020). Like the vertebrate midbrain, the pBV also receives input from other sensory systems, including, in the case of *Ciona*, the otolith and the peripheral nervous system. Moreover, the vertebrate midbrain is an important site of multisensory integration, and recent work from our laboratory has identified the pBV as a likely site for integration of visual and gravitaxis sensory inputs (Bostwick et al., 2020). The results presented here identifying the pBV as a likely site of FCD in visual processing, further supports the connections between the pBV and the midbrain. The combination of anatomical, gene expression, and connectivity data all point to a common origin for the pBV and the vertebrate midbrain. The alternative, that these similarities are the product of convergence, would appear to be much more unlikely, particularly in light of amphioxus data which show that cephalochordates, the most basal chordate subphylum, and whose divergence preceded the tunicate/vertebrate split, have conserved midbrain visual processing centers (Takahashi and Holland, 2004; Lacalli, 2006; Suzuki et al., 2015). Because cephalochordates, such as amphioxus, are basal to both tunicates and vertebrates, the convergence theory would have to postulate that the apparent homologies observed in both tunicates and cephalochordates arose independently, or that tunicates lost the midbrain homolog, and then independently evolved a brain region with similar gene expression, connectivity and anatomical location to those in vertebrates and cephalochordates.

## Materials and Methods

### Animals

Wild-type *Ciona robusta* (a.k.a., *Ciona intestinalis* type A) were collected from Santa Barbara Harbor. The animals carrying the mutation *pristine* (Salas et al., 2018) were cultured at the UC Santa Barbara Marine Lab (Veeman et al., 2011). Larvae were obtained by mixing dissected gametes of three adults and then culturing in natural seawater at 18°C. Homozygous *prs* larvae were produced by natural spawning of heterozygous or homozygous *prs* adults. For Figure 1C, two stable transgenic lines, vgat > kaede and vacht > CFP [provided by Y. Sasakura], were crossed to yield offspring with labeled GABAergic/glycinergic cells and cholinergic cells, respectively.

### Hybridization Chain Reaction (HCR) *in situ* and Immunolabeling

Whole mount fluorescent *in situ* hybridization of embryonic or larval *C. robusta* were performed using the hybridization chain reaction method (v. 3.0, Molecular Instruments; Los Angeles, CA, United States), as previously described (Kourakis et al., 2019). Complementary RNA probe sets were designed to coding regions for the following *Ciona* genes (unique gene identifiers provided in parentheses): *Otx* (NM_001032490.2), *en* (KH2012:KH.C7.431.v1.A.SL1-1), *pax3/7* (KH2012:KH.C10.150.v1.A.SL1-1), *AMPA receptor* (XM_018817034.1), *ADRα2a* (XP_018668148), *VGAT* (NM_001032573.1), and *pitx* (KH2012:KH.L153.79.v1.A.SL2-1). In larvae which underwent both *in situ* labeling and immunostaining, the *in situ* hybridization was performed first, followed by the immunolabeling (see below), after a transition from 5× SSCT to PBST.

Larvae for immunostaining were dechorionated at mid-tailbud stage using sodium thioglycolate, as for *in situ* hybridization, so that left-right asymmetric properties of the CNS would not be disrupted (Yoshida and Saiga, 2008). The immunostaining followed previously described procedures for *Ciona* (Newman-Smith et al., 2015). A primary antibody against *C. robusta* arrestin (Horie et al., 2005), raised in rabbit, was used at a dilution of 1:1,000. A secondary antibody, α-rabbit AlexaFluor 594 (Invitrogen; Waltham, MA, United States), was also used at 1:1,000. For vgat > kaede and vacht > CFP larvae, rabbit α-Kaede (MBL; Woburn, MA, United States) and mouse α-GFP (Life Technologies; Carlsbad, CA, United States) antibodies were used at 1:1,000, followed with appropriate AlexaFluor secondaries (Life Technologies), also at 1:1,000 dilution (described above).

Labeled animals (either by *in situ* or immunohistochemistry) were imaged on an Olympus Fluoview 1000 confocal microscope; post-image analysis used Imaris v6.4.0.0 or ImarisViewer v9.5.1 as well as Fiji (ImageJ) v. 2.0.0-rc-69/1.52p. The surface model depicted in Figure 1C was generated in Imaris v6.4.0.

### Behavioral Assays

All larvae were between 25 and 28 h post fertilization (hpf) (18°C). Larval swimming behaviors were recorded in sea water with 0.1% BSA using 10 cm agarose-coated petri dishes to reduce sticking. Image series were collected using a Hamamatsu Orca-ER camera fitted on a Navitar 7000 macro zoom lens. Programmable 700 and 505 nm LED lamps (Mightex) mounted above the petri dishes were used for dimming response assays as described previously (Kourakis et al., 2019; Bostwick et al., 2020). The dim response, adaptation, and reaction time movies were recorded at 5, 8.9, and 50 frames per second (fps), respectively. In the standard assay larvae were recorded for 10 s at the initial intensity (lux_0_) that was then dimmed (lux_F_) to specific values while image capture continued for 2 min. Larvae were allowed to recover for 5 min before being assayed again. All light intensity readings were taken with an Extech Instruments light meter.

### Drug Treatments

(*RS*)-AMPA hydrobromide (Tocris; Bristol, United Kingdom) was dissolved in filtered sea water to a stock concentration of 7.5 mM and then diluted to a final concentration of 500 μM. Dexmedetomidine (Tocris) was dissolved in filtered sea water to a stock concentration of 6.75 mM and then diluted to a final concentration of 20 μM. Larvae were incubated with the drug for about 10 min before beginning assays and remained in the drug solution through the entirety of the assay.

### Behavioral Quantification

Larvae with short bouts of swimming (<3 s) were not scored (Kourakis et al., 2019; Bostwick et al., 2020). Swim times, speeds, and tortuosities were calculated using the MATLAB script ELIANE (Kourakis et al., 2019). In the test for absolute adaptation (Figure 2), to measure the percent of larvae swimming at 1 s time intervals, the ELIANE script was modified to determine if a centroid (i.e., larva) in frame x was in the same position in frame x + 1. The sum of moving centroids was then divided by the total number of centroids. Percent of larvae that responded to dimming stimuli was quantified manually. R-squared values and Bayesian information criterion (BIC) were calculated using the program R. Larvae that had stuck to the petri dish, or that swam to the edges or into other larvae were not scored. Stuck larvae were defined as those that showed tail beating behavior, but did not move during the entire movie.

### Statistical Analyses

*T*-test was used to test significance between percent response (i.e., Figure 2D). Wilcoxon rank-sum test was used to pair-wises tests of significance (i.e., wild-type 10-fold vs. wild-type 60-fold). The Kruskal-Wallis test was used for testing significance of whole populations (see *p*-values in Figures 2A–C,F). Finally, Friedman’s test was used for comparison of larval groups in the FC series (i.e., wild-type vs. *prs*, control vs. AMPA).

## Data Availability Statement

The raw data supporting the conclusions of this article will be made available by the authors, without undue reservation.

## Author Contributions

CB and MK contributed to experimental design, data collection and analysis, and manuscript preparation. SS and LB contributed to data collection and analysis. WS contributed to research funding, experimental design, data collection and analysis, and manuscript preparation. All authors contributed to the article and approved the submitted version.

## Conflict of Interest

The authors declare that the research was conducted in the absence of any commercial or financial relationships that could be construed as a potential conflict of interest.

## Publisher’s Note

All claims expressed in this article are solely those of the authors and do not necessarily represent those of their affiliated organizations, or those of the publisher, the editors and the reviewers. Any product that may be evaluated in this article, or claim that may be made by its manufacturer, is not guaranteed or endorsed by the publisher.
