# Supplementary material for: Fold Change Detection in Visual Processing
Source: Front Neural Circuits. 2021 Aug 23;15:705161. doi: 10.3389/fncir.2021.705161 (PMC8419522; doi:10.3389/fncir.2021.705161)
Supplement: Supplementary file 3 [file Data_Sheet_1.PDF]

SFigure 1. Expanded data for Figure 2. A. Plot of data from Figure 2A showing other curves tested: log and linear. **B**. Plot of data from Figure 2F showing other curves tested: log and power. **C**. Number of larvae (n) assessed in the fold change series (3 - 60, first column) for WT swim time (panel 2A), speed (panel 2B), tortuosity (panel 2C), percent responding (panel 2D), and prs swim time (panel 2F). **D**. The experimental averages and standard deviations for the assays described in panel A. **E.** Pairwise tests for significance at each fold-change for swim time (WT and *prs*), speed, percent response and tortuosity (p-values shown; all comparisons by Wilcoxon, except percent response, which was done by T-test). Also shown (bottom right) is a comparison of swim times in WT and *prs* larvae at each fold-change step. p-values for each comparison are listed. WT = wild type; *prs* = *pristine* mutant; FC = fold change.

SFigure 2. Expanded data for Figure 3. A. Average swim times, standard deviations, and number of larvae analyzed for data presented in Figure 3A. **B**. Average swim times, standard deviations, and number of larvae analyzed for data presented in Figure 3B. **C**. Data for results plotted in Figure 3C. Sample size (n), percent of larvae responding, and test of significance (p-value; T-test) are shown. **D**. Data for results plotted in Figures 3E and F. Included in the table are the number of larvae analyzed at each dim, the average swim times (and standard deviations). Statistical analyses indicate that fewer larvae responded to Dim 2, but the average swim times were not different. **E.** Average response times, standard deviations, and number of larvae analyzed for data presented in Figure 3G. **F.** Pairwise statistical analysis of response times for results shown in Figure 3G (p-values shown; all comparisons by Wilcoxon).

SFigure 3. Expanded data for AMPA treatments shown in Figure 4. A. Averages of percent of control and AMPA-treated larvae responding to the indicated fold dimming for results plotted in Figure 4B. **B**. p-values (T-test) for pairwise comparisons at each fold change for results plotted in Figure 4B. **C.** Sample sizes (n), average swim times and standard deviations for data plotted in Figure 4C. **D**. Statistical analyses for data plotted in Figure 4C. All pairwise comparisons were made within treatments (AMPA or control) for each fold change (FC). p-valves are shown (Wilcoxon). **E.** Comparison of swim times at each fold change between treatments for results plotted in Figure 4C. p-valves are shown (Wilcoxon). **F.** Sample sizes (n), averages and standard deviations for results plotted in Figure 4D. **G**. Statistical analyses of swim times of AMPA-treated larvae for data plotted according to fold change (Figure 4D; left panel). All tests within a fold change group, and between fold changes, were performed by Wilcoxon (p-values shown). **H.** Statistical analyses of swim times of AMPA-treated larvae for data plotted according to initial light intensity (Figure 4D; right panel). The initial light intensity (before dim) and are indicated as 30 lux, 300 lux and 300 lux in the figure. All tests within an initial intensity group, and between initial intensities, were performed by Wilcoxon (p-values shown).

SFigure 4. Dexmedetomidine treatment of wild-type larvae. **A.** Swim times for control (Con) and dexmedetomidine-treated (Dex) larvae following 3-, 10- and 60-fold dimming. The initial (lux_0) and final (lux_F) illumination conditions are indicated. **B**. Response of dexmedetomidine-treated wild-type larvae to fold-change dims of different magnitudes. **C.** Data

from panel B sorted by magnitude of initial illumination (lux_0). **D**. Sample sizes (n), averages and standard deviations for results plotted in panels A-C. **E.** Statistical analyses of swim times of dexmedetomidine-treated larvae for data plotted according to fold change (panel B). **F.** Statistical analyses of swim times of dexmedetomidine-treated larvae for data plotted according to initial light intensity (panel C). The initial light intensity (before dim) and are indicated as 30 lux, 300 lux and 300 lux in the figure. **G.** Statistical analyses of swim times of dexmedetomidine-treated larvae versus non-treated for data plotted in panel A. All tests were performed by Wilcoxon (p-values shown). (* = $p < 0.05$; ** = $p < 0.01$; *** = $p < 0.001$; n.s. = not significant). For panels A-C, all data points (blue circles) and averages (red circles) are shown.

SFigure 5. Expanded data for Dexmedetomidine treatment of *pristine* larvae in Figure 4. **A**. Sample sizes (n), averages and standard deviations for control pristine results shown in Figure 4F. **B**. Sample sizes (n), averages and standard deviations for dexmedetomidine-treated pristine results shown in Figure 4F and G. **C.** Statistical analyses of swim times of dexmedetomidine-treated pristine larvae for data plotted according to fold change (Figure 4G). **D.** Statistical analyses of swim times of dexmedetomidine-treated pristine larvae versus non-treated for data plotted in Figure 4F. All tests were performed by Wilcoxon (p-values shown).

Movie 1. Response of *Ciona* larvae to 3-, 10- and 60-fold dimming. Larvae in 60 mm petri dishes are recorded from above. All larvae were initially exposed to a 505 nm LED lamp at 600 lux. The lamp was then dimmed to 200, 60 or 10 lux, as indicated. The swimming behavior of 10 larvae from each group is tracked (yellow boxes). The swim track is projected across time in color, and the yellow boxes disappear when the larvae stop swimming. Notice that the swims are progressively longer as larvae are exposed to the larger fold changes (*i.e.*, the projected swims in color are longer). The larvae were recorded using a far red light (700 nm). Movie plays at normal speed. Light dimming is evident at 5 seconds.

Movie 2. Absolute adaptation. Larvae are exposed to two dimming events separated by 2 minutes. The first dim is from 3000 to 300 lux, and the second from 300 lux to 30. Notice that swims are induced at each dimming, but that the swimming largely stops between dims. Video plays at 5X normal speed.

SFigure 1

A

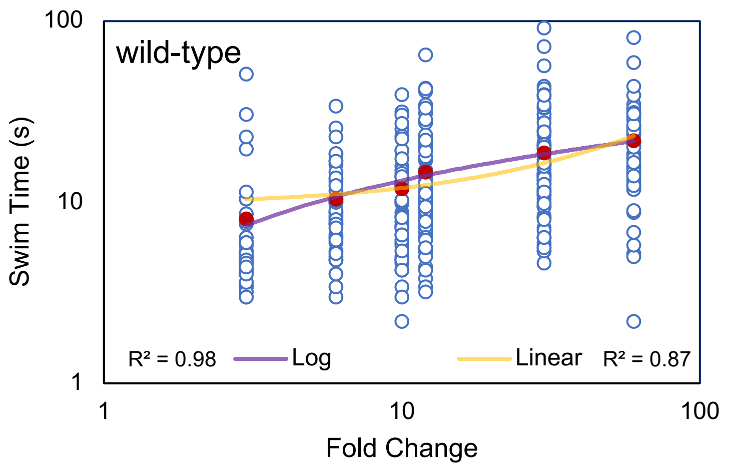

B

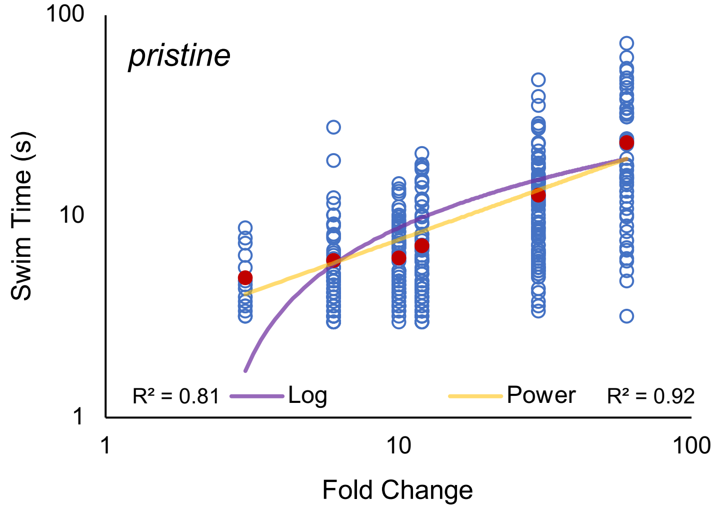

C

Experimental *n*-values

Fold Change	WT Swim Time	*prs* Swim Time	Speed	Tortuosity	% Respond
3	38	15	29	29	127
6	44	56	36	36	102
10	53	70	35	35	105
12	68	72	36	36	125
30	79	90	41	41	132
60	48	56	20	20	143

D

Experiment Averages ± Standard Deviation

Fold Change	WT Swim Time	*prs* Swim Time	Speed	Tortuosity	% Respond
3	8.07±9.15	4.97±1.82	1.56±0.99	4.75±14.69	60.06±5.95
6	10.45±6.30	6.07±4.22	1.40±0.98	3.12±14.27	81.94±9.89
10	11.88±7.95	6.24±2.78	1.65±0.96	4.30±13.85	96.16±4.01
12	14.63±11.38	7.19±4.40	1.41±0.94	4.70±13.48	94.61±4.06
30	18.70±14.80	12.84±8.26	1.51±0.92	4.14±13.15	100.00±0.00
60	21.86±13.98	23.30±15.99	1.42±0.90	6.07±12.82	99.19±1.41

E

Statistical Test *p*-values

WT Swim Times

FC	3	6	10	12	30
6	4.29E-04				
10	2.79E-04	0.2665			
12	1.41E-05	0.0223	0.1093		
30	1.34E-07	1.78E-05	2.67E-04	0.0088	
60	1.77E-07	2.17E-07	2.56E-06	1.18E-04	0.0194

Speed

FC	3	6	10	12	30
6	0.4095				
10	0.8292	0.2206			
12	0.2222	0.7740	0.2385		
30	0.5197	0.7323	0.5048	0.6867	
60	0.1895	0.7517	0.2446	0.5901	0.4024

prs Swim Times

FC	3	6	10	12	30
6	0.2721				
10	0.035	0.0565			
12	0.0464	0.0446	0.3559		
30	1.09E-06	7.99E-12	1.74E-11	1.12E-08	
60	5.89E-08	1.46E-14	8.21E-16	1.04E-13	5.85E-06

% Respond

FC	3	6	10	12	30
6	0.0304				
10	0.0010	0.0823			
12	0.0011	0.1096	0.6618		
30	0.0003	0.0342	0.1729	0.0832	
60	0.0004	0.0404	0.2854	0.1390	0.3739

Tortuosit
y

FC	3	6	10	12	30
6	0.7665				
10	0.6563	0.5011			
12	0.7365	0.5849	0.9587		
30	0.2379	0.4717	0.1595	0.1269	
60	0.5085	0.9932	0.6058	0.5668	0.6727

Fold-Change	WT vs. PRS
3	0.6642
6	2.05E-06
10	9.94E-07
12	5.14E-08
30	0.0018
60	0.8962

SFigure 2

A

Wild-type

Fold Change	3-fold			10-fold			60-fold		
Δlux	3000to1000	300to100	30to10	3000to300	300to30	30to3	3000to50	300to5	30to0.5
Average ± SD	8.23±5.89	7.49±6.42	8.35±5.74	12.81±9.41	12.74±7.95	12.56±10.90	20.32±15.43	20.61±16.86	19.92±19.55
n	39	19	34	62	39	54	31	34	43

within fold changes

3-fold	3000to1000	300to100
300to100	0.7327	
30to10	0.3187	0.4726

10-fold	3000to300	300to30
300to30	0.7246	
30to3	0.2942	0.2705

60-fold	3000to50	300to5
300to5	0.2737	
30to0.5	0.8595	0.3729

between fold changes

	3-fold	10-fold
10-fold	1.88E-05	
60-fold	4.36E-11	2.79E-04

B

Pristine

Fold Change	3-fold			10-fold			60-fold		
Δlux	3000to1000	300to100	30to10	3000to300	300to30	30to3	3000to50	300to5	30to0.5
Average ± SD	11.30±14.91	8.81±8.19	4.78±1.59	8.00±7.09	9.31±7.14	9.25±5.31	17.68±13.26	31.02±17.29	19.65±12.78
n	22	26	12	28	19	34	5	10	23

within fold changes

3-fold	3000to1000	300to100
300to100	0.1818	
30to10	0.6781	0.0142

10-fold	3000to300	300to30
300to30	0.7946	
30to3	0.1788	0.5403

60-fold	3000to50	300to5
300to5	0.1292	
30to0.5	0.7414	0.0685

between fold changes

	3-fold	10-fold
10-fold	0.01977	
60-fold	1.40E-08	1.18E-08

C

Weber's Law			
	300to30	3000to2730	p-value
% Responded	88.04±4.94	4.10±2.82	1.00E-07
n	776	443	

D

Light Adaptation			
	Dim 1	Dim 2	p-value
Swim Time	8.47±5.24	6.72±3.27	0.4445
% Responded	87.25	64.29	2.28E-04
n	102	84	

E Experiment Data for response times

Fold-Change	Average ± SD	n
3	0.50±0.17	39
6	0.40±0.14	65
10	0.32±0.10	64
12	0.30±0.11	55
30	0.25±0.06	52
60	0.19±0.04	49

F Statistical Test p-values for response times

FC	3	6	10	12	30
6	3.80E-03				
10	2.56E-08	1.65E-04			
12	2.45E-08	5.39E-06	0.0701		
30	5.14E-12	2.51E-11	7.00E-06	0.009	
60	1.03E-14	1.91E-17	5.01E-15	2.30E-10	2.28E-07

SFigure 3

A **Percent Responding**: Experiment Averages ± Standard Deviation

Fold Change	Control	AMPA
3	69.15±9.69	80.51±12.88
6	85.08±9.64	84.73±5.41
10	87.81±9.20	91.85±4.73
12	92.49±4.57	95.77±5.06
30	95.04±5.07	97.16±1.24
60	94.51±5.94	99.59±0.70

B **Percent Responding**: p-values for pairwise comparisons

Fold Change	Control vs. AMPA
3	0.2890
6	0.9588
10	0.5360
12	0.4517
30	0.5201
60	0.2153

C **Swim Times**: Experiment Averages ± Standard Deviation

	AMPA		Control	
Fold Change	Swim Time	n	Swim Time	n
3	6.97±4.58	47	11.24±8.24	22
6	8.86±7.77	57	13.55±10.29	17
10	9.96±12.82	51	16.44±11	22
12	9.56±7.15	57	16.6±12.69	31
30	11.79±10.63	72	20.04±13.75	37
60	10.58±7.68	36	22.59±13.77	30

D **Swim Times**: Comparisons within treatments (p-values)

AMPA

FC	3	6	10	12	30
6	0.1340				
10	0.0680	0.3932			
12	0.0332	0.2563	0.3343		
30	0.0023	0.0343	0.0620	0.1397	
60	0.0089	0.0801	0.1145	0.1971	0.5570

Control

FC	3	6	10	12	30
6	0.3100				
10	0.0422	0.1977			
12	0.0216	0.1883	0.5144		
30	5.11E-04	0.0181	0.1775	0.0561	
60	1.36E-04	0.0051	0.0515	0.0122	0.1737

E **Swim Times**: Comparisons between treatments (p-values)

AMPA vs Control	
3-fold	0.0074
6-fold	0.0257
10-fold	4.30E-03
12-fold	5.40E-04
30-fold	2.54E-05
60-fold	1.46E-05

F

Fold Change	3-fold			10-fold			60-fold		
Δlux	3000to1000	300to100	30to10	3000to300	300to30	30to3	3000to50	300to5	30to0.5
Average ± SD	12.56±12.18	10.55±9.08	8.31±10.78	17.49±14.76	10.46±7.11	6.60±4.44	12.28±10.59	8.82±6.07	9.43±7.21
n	14	12	27	14	32	54	15	37	45

G

within fold changes

3-fold	3000to1000	300to100
300to100	0.5035	
30to10	0.01491	0.1852

10-fold	3000to300	300to30
300to30	0.1931	
30to3	0.0009099	0.001755

60-fold	3000to50	300to5
300to5	0.785	
30to0.5	0.8376	0.8851

between fold changes

	3-fold	10-fold
10-fold	0.6773	
60-fold	0.4178	0.5278

H

within orders of magnitudes

**30lux**	30to0.5	30to3
30to3	0.02465	
30to10	0.04918	0.7712

**300lux**	300to5	300to30
300to30	0.3034	
300to100	0.7891	0.6732

**3000lux**	3000to50	3000to300
3000to300	0.2384	
3000to1000	0.6004	0.2801

between order of magnitudes

	**30lux**	**300lux**
**300lux**	0.006626	
**3000lux**	0.001368	0.1755

SFigure 4

A

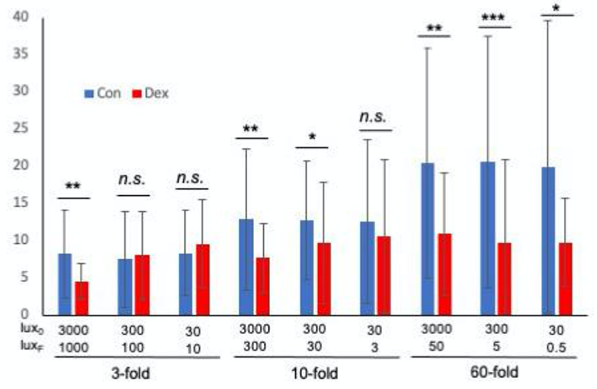

B

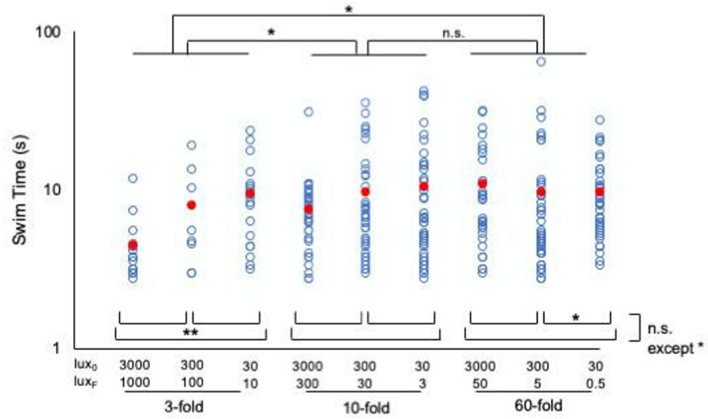

C

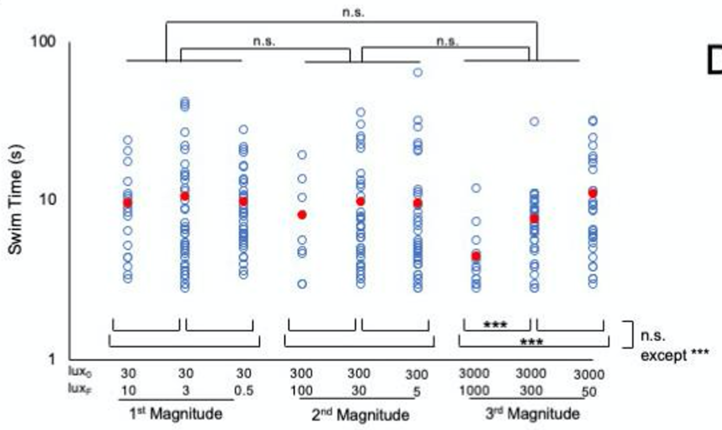

D

Fold Change	3-fold			10-fold			60-fold		
Δlux	3000to1000	300to100	30to10	3000to300	300to30	30to3	3000to50	300to5	30to0.5
Average ± SD	4.49±2.36	8.03±5.86	9.57±5.93	7.64±4.69	9.76±8.13	10.59±10.18	10.92±8.19	9.66±11.21	9.74±5.86
n	15	8	18	37	38	41	29	42	35

E

within fold changes

3-fold	3000to1000	300to100
300to100	0.1116	
30to10	0.001536	0.4873

10-fold	3000to300	300to30
300to30	0.9746	
30to3	0.9362	0.8405

60-fold	3000to50	300to5
300to5	0.09424	
30to0.5	0.9946	0.03327

between fold changes

	3-fold	10-fold
10-fold	0.0405	
60-fold	0.01463	0.3241

F

within orders of magnitudes

1st Mag	30to0.5	30to3
30to3	0.1415	
30to10	0.4329	0.6923

2nd Mag	300to5	300to30
300to30	0.8598	
300to100	0.4526	0.2126

3rd Mag	3000to50	3000to300
3000to300	0.1318	
3000to1000	2.3691e-04	6.3076e-04

between order of magnitudes

	1st Mag	2nd Mag
2nd Mag	0.1167	
3rd Mag	0.0973	0.9799

G

treated vs. untreated

Fold Change	3-fold			10-fold			60-fold		
Δlux	3000to1000	300to100	30to10	3000to300	300to30	30to3	3000to50	300to5	30to0.5
p-value	0.0013	0.6703	0.2897	0.0013	0.0196	0.266	0.0036	3.67E-05	0.034

SFigure 5

A

Untreated *prs*

Fold Change	3-fold			10-fold			60-fold		
Δlux	3000to1000	300to100	30to10	3000to300	300to30	30to3	3000to50	300to5	30to0.5
Average ± SD	11.30±14.91	8.81±8.19	4.78±1.59	8.00±7.09	9.31±7.14	9.25±5.31	17.68±13.26	31.02±17.29	19.65±12.78
n	22	26	12	28	19	34	5	10	23

B

Dexmedetomidine-treated *prs*

Fold Change	3-fold			10-fold			60-fold		
Δlux	3000to1000	300to100	30to10	3000to300	300to30	30to3	3000to50	300to5	30to0.5
Average ± SD	10.20±8.80	4.20±2.00	9.30±9.19	3.16±0.72	6.67±8.03	4.69±1.52	7.46±5.32	9.97±8.51	6.11±2.39
n	5	4	2	9	18	11	25	26	30

C

within fold changes

10-fold	3000to300	300to30
300to30	0.9988	
30to3	0.9947	0.3849

60-fold	3000to50	300to5
300to5	0.9663	
30to0.5	0.3739	0.0078

between fold changes

10-fold vs. 60-fold
p = 0.00000474

D

treated vs. untreated

Fold Change	10-fold			60-fold		
Δlux	3000to300	300to30	30to3	3000to50	300to5	30to0.5
p-value	0.000104	0.0344	0.0019	0.0122	0.000840	3.95E-06
